# A Case Report on Stroke in a Young Patient: Looking Beyond the Obvious Cause

**DOI:** 10.7759/cureus.90555

**Published:** 2025-08-20

**Authors:** Sowmitra Das, Farhad Uddin Khan, Humayra Tabassum, Aklima Akter, Tangila Akter, Khondakar Asiful Kabir

**Affiliations:** 1 Internal Medicine, King's College Hospital NHS Foundation Trust, London, GBR; 2 General Medicine, King's College Hospital NHS Foundation Trust, Orpington, GBR; 3 Internal Medicine, King's College Hospital NHS Foundation Trust, Orpington, GBR

**Keywords:** ct head angiography, horners syndrome, nihss score, spontaneous internal carotid artery dissection, stroke, transient ischemic attacks, ­trauma

## Abstract

Carotid artery dissection is a condition in which the layers of the carotid artery are separated. As a result, blood flow to a particular region of the brain is interrupted. The condition can occur extra-cranially or intra-cranially and can lead to subarachnoid hemorrhage or ischemic stroke, and it is, in fact, one of the most common causes of stroke in young patients. The considerable variation in presentation makes carotid artery dissection difficult to diagnose early. This case report describes the presentation of internal carotid artery (ICA) dissection leading to stroke in a young patient.

## Introduction

Carotid artery dissection is common in all age groups and accounts for 2.5% of all strokes, including those in individuals under 40 years of age [1]. In young patients, 20% of cerebrovascular diseases are caused by carotid artery dissection, with a slightly higher incidence in males [2]. Carotid artery dissection occurs when a tear in the intimal layer of the carotid artery, whether spontaneous or caused by trauma, creates an intramural hematoma [1]. The intramural hematoma causes stenosis and, eventually, the formation of a thrombus [3]. Traumatic dissection can occur as a result of either blunt or penetrating trauma. The blunt trauma can be significant or seem minimal (chiropractic manipulation being a classic example). For example, a motor vehicle accident may involve rapid deceleration with simultaneous neck hyperextension and rotation that leads to an intimal tear of the carotid artery. However, most spontaneous carotid dissections are idiopathic, and a family history of dissection significantly increases an individual’s risk, so caution is crucial [2]. Marfan syndrome, Ehlers-Danlos syndrome, fibromuscular dysplasia, and other connective tissue disorders also increase the risk of carotid artery dissection [1-3].

## Case presentation

A 40-year-old man presented to the emergency department (ED) after having experienced blurred vision for 60 minutes, numbness on the right side of his body, and the inability to speak for five to 10 minutes. He started to develop these symptoms while returning from work in the afternoon. By the time of assessment in the ED, the symptoms had resolved apart from some transient numbness on the right side of his face. No significant past medical history was noted other than a panic attack three years previously, and there was no history of stroke in his family. The patient said that he had not experienced any trauma to the head, neck, or any limb or any facial weakness. He maintained a healthy lifestyle and had started weightlifting in the gym three to four weeks previously.

**Figure 1:**
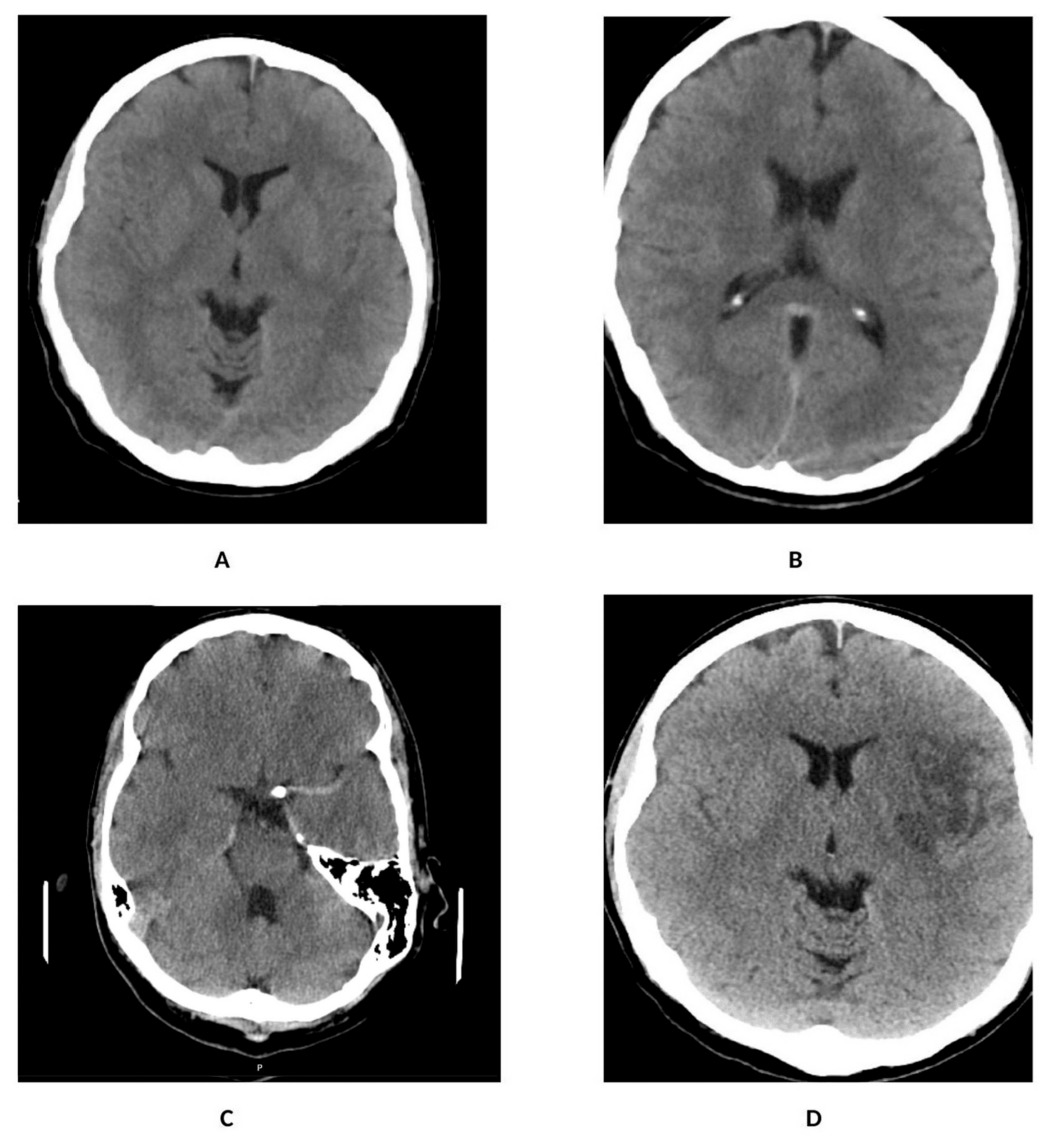
A: CT scan of the head on admission showing no acute findings. B: Second CT scan after the initial weakness resolved, showing no definite intracranial pathology. C: Evidence of a dense left MCA sign with corresponding occlusion of the left MCA (M1 segment). D: CT scan of the head post-thrombectomy showing an established infarct in the left MCA territory CT: computerized tomography; MCA: middle cerebral artery

**Figure 2:**
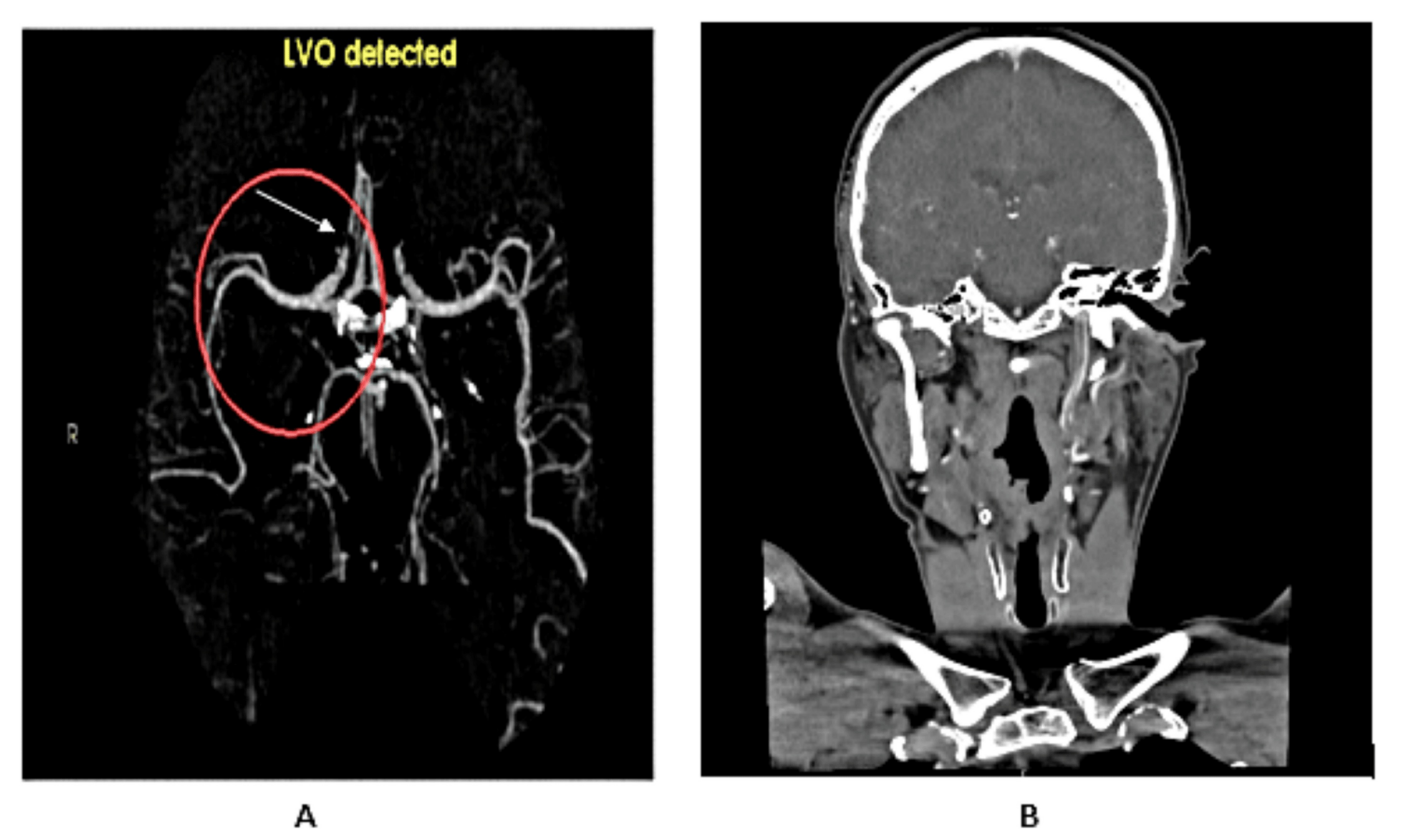
CT angiography of the head and neck showing a large filling defect noted in the left ICA extending 63 mm from its origin to the carotid canal and the base of the skull. These features are highly suggestive of an ICA dissection CT: computerized tomography; ICA: internal carotid artery

On initial assessment, his National Institutes of Health Stroke Scale (NIHSS) score was 0. A computed tomography (CT) scan of the head and CT angiography of the brain showed a false lumen in the left internal carotid artery (ICA) extending 63 mm from its origin and reaching the carotid canal and the base of the skull (Figures 1, 2).

These findings are highly suggestive of ICA dissection. Conservative management was planned based on the neurological assessment. However, the patient began to feel unwell and started sweating profusely as the results of his scan were being explained to him in the ED. On review, weakness in the right arm and right lower face and slurring of speech were noted. A neurological assessment revealed an NIHSS score of 6, specifically, 2 for partial right lower face paralysis, 2 for right arm weakness, and 2 for severe dysarthria. Some degree of cognitive involvement was also found, demonstrated by the patient’s inability to state his age or the month. Repeat CT head and CT angiography of the brain showed no interval changes, and the findings remained consistent with a diagnosis of ICA dissection.

Before rescanning, thrombolysis was planned based on the NIHSS score of 6. However, after returning from the scan, the patient’s symptoms started to improve. His arm weakness decreased significantly, and his NIHSS score decreased to 3, allowing for a shift to conservative management. Priority was given to monitoring in the Hyper Acute Stroke Unit for 24 hours.

Unfortunately, the next morning, the patient was found on the floor and, unable to stand up, was trying to pull himself to the bed. Members of the ward staff confirmed that he had been doing well around 10 minutes previously, was self-caring, and had eaten breakfast independently. Upon immediate assessment, his NIHSS score was 20, and he was experiencing obvious dense right-sided weakness, the inability to follow commands, a deviated gaze to the left, and the absence of visual threat to the right. A Glasgow Coma Scale of 11 (eye-opening 4, verbal response 2, motor response 5) was noted.

CT head and CT angiography of the brain were repeated, and the results revealed a left middle cerebral artery (M1) occlusion and an ICA thrombus secondary to the dissection. After ruling out fractures following a fall, our neurology team swiftly proceeded with thrombolysis. The case was then discussed with the interventional neuroradiologist, and the decision was made to perform mechanical thrombectomy (MT). Before the procedure, the patient was re-evaluated, and his NIHSS score had improved to 15 post-thrombolysis.

MT was successful, and a stent was placed in the ICA, which was found to be narrowed during the procedure. Before the procedure, the expanded thrombolysis in cerebral infarction score was -1, and the score after the procedure was -3. No peri-procedural issues were reported by the anesthetist. Postoperatively, the patient was recovering well and was extubated as planned. The NIHSS score had improved to 5, and the plan was to start dual antiplatelet therapy (DAPT) and continue it for six months because of the high risk of stent reocclusion. Postoperative CT head showed intra-parenchymal changes in the left middle cerebral artery territory consistent with micro-hemorrhagic transformation following MT without significant mass effect. The scan was reviewed by a neuroradiology multidisciplinary team, and it was agreed that DAPT would be continued. Investigations conducted to identify secondary causes of stroke yielded negative results (Table 1).

**Table 1:** Routine bloods, including antibody test results to identify secondary causes of stroke PT: prothrombin time; INR: international normalized ratio; APTT: activated partial thromboplastin time; eGFR: estimated glomerular filtration rate; CKD-EPI: chronic kidney disease epidemiology; MPO: myeloperoxidase; PR3: proteinase 3; IU: international unit

Test	Result	Reference range
White cell count	10.3	4.0-11.0/L
Hemoglobin	134	130-165 g/L
Platelet count	302	(150-450) 10^9^/L
Neutrophils	8.87 (H)	(1.50-6.30) 10^9^/L
PT	10.6	10.0-12.0 sec
INR	1.0	0.9-1.2 ratio
APTT	24.0	20.0-29.0 sec
APTT (ratio)	0.9	0.8-1.1 ratio
Sodium	140	135-145 mmol/L
Potassium	3.8	3.5-5.3 mmol/L
Creatinine	69	61-123 µmol/L
eGFR by CKD-EPI (2009)	>90	NA mL/min/1.73m^2^ >90
Free protein S antigen	91	60%-150%
Homocysteine	<15	<15.0 µmol/L
Protein C activity	103.8	70.0-140.0 IU/dL
Antithrombin activity IIa	109.6	80.0-130.0 IU/dL
Anti-MPO antibodies	<0.2	<3.5 IU/mL
Anti-PR3 antibodies	<0.2	<2.0 IU/mL
Connective tissue disease	0.1	0.0-0.6 ratio

The patient recovered well, thanks to the excellent outcome of the MT, and was discharged with a follow-up in the stroke clinic. He was advised to avoid heavy lifting, quit smoking, and not drive until the next follow-up. During follow-up, the patient was found to be committed to his recovery, being compliant with the medications and advice. He had completed six months of DPAT and was placed on maintenance.

A follow-up Doppler scan three months later showed a patent stent in the proximal aspect of the ICA with normal flow. In the meantime, echocardiography had excluded any cardiac thrombus, and 24-hour Holter monitoring showed sinus bradycardia along with first-degree heart block. In a telephone consultation six months later, the patient’s partner reported significant improvements in his condition, including his speech and normal arm movements. He remained compliant with his medication and had successfully quit smoking.

## Discussion

The management of carotid artery dissection depends on multiple factors, including the location and cause. In conservative management, antiplatelet agents play a significant role in preventing further stroke. However, a significant number of the patients also require an endovascular stent [4,5]. All carotid artery dissection patients have a very high risk of stroke and intracranial bleeding and are subject to anticoagulation, which also involves risks [2].

We have presented here a case of a young man with transient and recurrent episodes of neurological symptoms without any significant risk factors and a low initial assessment score, along with negative findings in the CT scans. Repeat imaging after a third episode established a diagnosis of stroke secondary to an ICA dissection. The patient underwent thrombolysis and MT with a stent in the ICA. Upon completion of the suggested DAPT, the patient’s symptoms showed significant and persistent improvement.

## Conclusions

Our goal in presenting this case report is to raise awareness among clinicians about the varied presentations and to emphasize the importance of clinical suspicion in achieving a prompt and accurate diagnosis. While stroke secondary to ICA dissection following thrombectomy has a good prognosis, it is essential to ensure adequate compliance with treatment and conduct proper follow-up. This commitment to follow-up care is a key part of clinicians’ responsibility to their patients.
